# Enhancing digital twin performance through optimizing graph reduction of finite element models

**DOI:** 10.1038/s41598-025-20571-z

**Published:** 2025-10-29

**Authors:** Marek Ciklamini, Matous Cejnek

**Affiliations:** Department of Instrumentation and Control Engineering, Czech Technical University in Prague, Technicka 4, 16607 Prague, Czechia

**Keywords:** Graph reduction, Nodes regression, Digital twin, Hybrid modeling, Finite element model, Mechanical engineering, Design, synthesis and processing

## Abstract

This research compares Graph Neural Networks reduction techniques for computational modeling in structural engineering. The proposed Finite Element Shape Logic Graph technology serves as a benchmark, capturing intricate details through fully connected logic graphs derived from Finite Element Models. Multiple graph reduction methods are introduced, each addressing computational efficiency: shortest path connectivity, Laplacian Matrix for Dimensionality Reduction, and an approach exploiting graph sparsity. This research contributes to structural engineering and offers insight for diverse applications in the evolving landscape of digital twins, particularly within the specific context of graph optimization and the efficiency of physical neural networks. Our proposed techniques are tested on datasets consisting of four different finite element models. Our study suggests that, while the shortest path method is a promising starting point for graph reduction in mechanical system modeling, leveraging Laplacian spectral reduction techniques is essential for achieving optimal solutions, particularly for larger finite element models. This approach served as the basis for the development of digital twins of mechanical structures, ensuring accurate representation and efficient analysis of complex engineering systems.

## Introduction

In the dynamic landscape of structural engineering and digital twinning, the quest for precision and efficiency in computational modeling has led us to explore innovative methodologies, often by exploiting well-established methodology, such as Finite element model (FEM)^3^. FEM is an indispensable tool for simulating complex mechanical systems over 80 years. However, as the complexity of the required models grows, so does the computational demand on classical FEM, prompting a paradigm shift in our approach.

Interdisciplinary collaboration plays a pivotal role in pushing the boundaries of FEM-based computational modelling, particularly in addressing complex engineering challenges. By bringing together experts from various fields, such as finite element analysis, data science, mechanical engineering, and computer science, interdisciplinary teams can take advantage of the diverse perspectives and expertise to tackle multifaceted problems more effectively. In FEM-based computational modelling, a collaboration between experts in finite element analysis and data science allows the integration of advanced data-driven techniques into traditional modelling approaches. This integration enables more accurate and efficient simulations by leveraging large datasets, machine learning algorithms, and advanced computational methods. A crucial piece of this evolution is the ability to tailor and modify classical methods so that they better align with emerging data-driven paradigms. Rather than replacing FEM entirely, recent work explores hybridization—physics-informed neural networks that embed governing equations directly into the learning process. These models illustrate how physical representation can be utilized while allowing flexible adaptation to new data or changing boundary conditions. Similarly, digital shadowing approaches \cite{DigitalShadowBuilding} demonstrate how physical models are fruitfully adapted in real time, tailoring their fidelity to the level of information available from sensors or monitoring systems. This principle of modification where models are continuously adjusted rather than statically predefined represents a connecting chain in the shift from engineering-focused digital twins to their adoption in medicine. In surgical contexts, digital twins are already showing promise as adaptive, personalised training and decision-support tools, paving the way for multimodal AI systems that integrate imaging, simulation, and real-world data into a single adaptive framework. Beyond individual domains, the tailoring and modification of digital twin and shadow frameworks must also be seen through the lens of digital transformation—where cross-domain interoperability, comparative analysis, and platform-based services become key enablers of large-scale adoption.

The proposed research approaches the challenge by harnessing the power of graph theory within the realm of FEMs. Graphs, extracted from FEMs, serve not only as visual representations but also as dynamic structural blueprints. These graphs become the foundation for training Graph Neural Networks (GNNs)^4^, introducing a learning component to the traditional modeling process. Therefore, this approach combines white box modeling with machine learning and has been successfully used in various domains, such as in the field of medical imaging, where GNNs were used to model soft tissue. In study^5^, the authors propose the use of a physics-driven GNN-based framework for processing high-quality images for surgical planning. The study is reporting results with errors lower than or similar to state-of-the-art approaches with affordable computational costs. Many other applications similarly exploit the benefits of GNN, leveraging their ability to incorporate mesh-based physics information^6^ into machine learning models, which are nowadays recognized as physics-performed machine learning^7^.

Utilizing graph theory principles enhances modelling capabilities compared to traditional FEM by providing a more flexible and scalable framework for representing complex structural systems in a sense, especially from model time response and knowledge encapturing. Graphs offer a more intuitive and natural representation of interconnected components within a system, allowing for a more comprehensive understanding of the relationships between different elements.

Graph reduction plays a crucial role in enabling real-time predictive modeling by significantly reducing the computational burden associated with FEMs while preserving essential structural information. Traditional FEM simulations are computationally expensive, especially for large-scale mechanical systems, limiting their use in real-time applications. By transforming FEMs into graph-based representations and applying graph reduction techniques, we streamline the data structure, eliminating redundant connections while maintaining the core physical relationships necessary for accurate predictions. This reduction not only accelerates the training and inference of GNNs but also enhances their scalability, allowing real-time applications such as structural health monitoring, failure prediction, and adaptive control in digital twins. Additionally, spectral reduction techniques, such as Laplacian matrix-based methods, enable the retention of dominant structural characteristics, ensuring that reduced models maintain predictive accuracy even in complex mechanical scenarios. By optimizing graph structures, we make it feasible to deploy lightweight, low-latency predictive models, allowing engineers to make data-driven decisions in real time without the computational overhead of full-scale FEM analysis. This integration of graph reduction with machine learning-based predictive modeling represents a transformative step towards fast, efficient, and adaptive digital twin systems.

The rise in scale and complexity of engineering projects raises the challenge of computational intensity associated with detailed FEMs. It is within this challenge that our focus on graph reduction emerges. Through systematic simplification of these graphs, we aim not only to streamline computational complexity but also to lay the groundwork for the efficient training of GNNs.

At the heart of our methodology lies the integration of Graph Neural Networks. These neural networks, trained on data sets derived from FEM graphs and nodal inputs, transcend the traditional boundaries of simulation. By performing regression on nodes. The GNNs learn and generalize the intricate relationships embedded in the structural graphs, enabling them to predict behaviors beyond the scope of conventional modeling.

The synergy between graph reduction and GNNs is pivotal in our journey towards an accurate digital twin. As we distill complex structural representations into reduced graphs, we simultaneously empower our neural networks to learn and predict with unprecedented accuracy. This dual approach not only optimizes computational efficiency but also establishes a robust framework for real-time predictive modeling.

To summarize the proposed methodology, it can be broken down in steps as follows:

1. Creation of FEM,
2. Replacement of FEM by digital twin (GNN),
3. Reduction of digital twin complexity.

The quality of the reduced digital twin is tested against the original GNN. Therefore, in this study, we call the original trained GNN a benchmark. In the subsequent sections, it is proposed methodology of training process of FEM to GNN conversion and the following GNN pruning. Our findings, based on computational experiments, not only hold promise for structural engineering but extend into diverse applications within the evolving landscape of digital twins.

To our knowledge, there are no studies of the graph reduction techniques aiming to significantly decrease digital twinning of mechanical structures.

### Bridging physical systems and graph-based digital models

In digital twin modelling, a clear distinction must be made between the physical and digital spaces, as both play distinct yet interconnected roles. The physical space consists of the real-world mechanical structure, where physical components such as beams, trusses, or aircraft wings experience actual loading conditions, external forces, and material deformations. These physical systems are monitored using sensors that collect real-time operational data, including strain, displacement, and stress distribution. In contrast, the digital space represents a computational abstraction of the physical system, often in the form of a graph-based digital twin. Here, FEMs are transformed into graph structures, where nodes correspond to discrete physical points, and edges capture structural connectivity. GNNs leverage this representation to simulate and predict mechanical responses under varying conditions, providing an efficient alternative to traditional FEM simulations. Additionally, graph reduction techniques are employed to simplify these models while retaining their essential features, thereby optimizing computational efficiency. The interaction between these two spaces is critical-data from the physical space continuously updates the digital twin, refining its predictive accuracy, while insights from the digital model inform real-world decisions such as failure prevention and performance optimization. This synergy ensures that the digital twin remains an accurate and computationally efficient counterpart to its physical counterpart, enabling real-time monitoring and adaptive modeling of complex mechanical structures.

## Materials and methods

In this section we introduce the proposed approach, the dataset used, and the experimental design.

### Graph extraction from FEM

The GNN is employed to train a bidirectional graph extracted from the FEM structure using algorithms presented in the appendices. This fully connected graph reflects the logical relationships defined by the finite-element shapes, as demonstrated in our previous work^1^, highlighting its effectiveness in capturing intricate structural details. Moreover, the structural character is identified without calculating the structure’s eigenmodes, shapes and eigenvectors, focusing solely on the static structural domain to be implemented as a Digital Twin.

Once the Finite Element Model is established, its geometric and domain description is documented through a computational environment, which defines the spatial configuration of each node $n_{i}$ within a particular element $e_{i}$, as well as the mutual connectivity between the elements. The graph model $\mathcal {G}$ can be obtained as follows:

1 $$\begin{aligned} f:\Omega (\Gamma (e,n),E,\nu ,F)\rightarrow \mathcal {G} \left( N, V \right) , \end{aligned}$$

where $\Omega$ represents the FEM model, $\Gamma$ describes the geometry of the structure, *E* defines the material properties, including Young’s modulus and Poisson’s coefficient $\nu$, *F* represents the applied forces and boundary conditions on the mechanical structure, *N* denotes the nodes, and *V* denotes the edges of the graph.

In capturing critical features and behaviours, the rationale behind involving all nodes lies in enhancing the fidelity and accuracy of the reduced representation of the mechanical system. Each node in the system contributes unique information about its local environment and interactions with neighbouring nodes. By involving all nodes in the reduction process, we ensure that all potentially crucial information is noticed and included. This comprehensive approach allows us to capture the full complexity of the system’s structure and dynamics, enabling the reduced representation to reflect the behaviour of the original mechanical system faithfully. Additionally, by incorporating information from all nodes, we can better preserve important structural characteristics and relationships, ensuring that the reduced representation accurately captures the system’s essential features.

The participation of all nodes enhances the fidelity and accuracy of the reduced representation; nodes and edges in the resulting graph adhere to the logic of the original FEM. The nodes acquire data from the converged FEM, with input parameters *X* capturing reaction forces $F_i$ of the selected nodes $n_i \in \mathcal {S}$ simulating distributed sensors. The target output $\textbf{y}$ represents structural mechanical stress for all nodes $\sigma$. Therefore, the graph can be described as follows:

2 $$\begin{aligned} \mathcal {G_{FEM}}: \left( N, V, \mathbf {X(F)}, \mathbf {y(\sigma )} \right) . \end{aligned}$$

With such a model, we aim to leverage the information available to each element of the mesh, which serves as a discretization of the entire geometric structure. The main idea revolves around utilising each node of the element as a node of the graph, establishing links between graph nodes based on the edges of the specified element. This graph extraction approach, rooted in the nodal structure and topology of the element, has been extensively explored^8^. There might exists several strategies for graph extraction, but we have opted to follow the element typology approach outlined in mentioned algorithms due to its intuitive meaning. This approach forms the foundation for subsequent analyses, particularly in the context of Laplacian matrix computation.

This initial description sets the stage for solving the physical models represented by partial differential equations within the specified domain. As part of this process, the Laplacian matrix of a graph $L \left( {\mathcal {G}} \right)$, derived standardly from the adjacency matrix *A* as a real symmetric matrix, with the degree matrix $D \left( A \right)$, plays a pivotal role. The Laplacian matrix *L* is a positive semidefinite matrix that reflects the connectivity and topology of the graph. It serves as a cornerstone for the spectral reduction strategy mentioned in more detail, encapsulating crucial structural information essential for subsequent analyses.

3 $$\begin{aligned} L \left( {\mathcal {G}} \right) = D \left( A \right) - A \left( {\mathcal {G}} \right) \end{aligned}$$

This study utilises and compares two different frameworks of graph neural network architectures. The following subsections describe both architectures.

#### Graph convolutional network

The following graph network architecture comprises a graph convolutional layer (GCN). The graph convolutional neural network^9^ can be understood as the enhancement of the Feed Forward Neural Network (FFNN) with classical notation^10^ and so by the graph-structured data described, taking into account the adjacency matrix *A*. The forward pass for the first hidden layer is then

4 $$\begin{aligned} H_{1}=\hat{\textbf{D}}^{-1/2} \cdot \hat{{A}} \cdot \hat{{D}}^{-1/2} \cdot \textbf{X} \cdot \textbf{W}_{0}+\textbf{b}_{0}, \end{aligned}$$

where the notation of message passing information through the graph is similar to forward propagation, well known in classical FFNN: $\textbf{W}$ is matrix of weights, input features from dataset $\mathbf {X_{0}}$ and bias vector $\textbf{b}_{0}$.

#### Sample and aggregated embeddings

Second approach we used is built on Sample and Aggregated Embeddings (SAGE). It is a type of neural network layer designed to aggregate information from neighbouring nodes in a graph structure^11^. By incorporating Sage layers, the promising target lies in capturing complex relationships and dependencies between nodes in the graph representation of the mechanical structure, providing a flexible and effective approach to graph modelling. In SAGE layers, the node embeddings of neighbouring nodes are computed and aggregated to generate a new node embedding. This aggregated embedding is then used to update the embedding of the target node, and the process is repeated until all nodes in the graph have been updated. As a type of GNN layer, SAGE layers aggregate information from a node’s neighbourhood to generate a node representation. The forward pass is expressed as

5 $$\begin{aligned} H_{1}=\left[ AGG\left( \textbf{X} \right) ||\textbf{X} \right] \textbf{W}_{0}+\textbf{b}_{0}, \end{aligned}$$

where *AGG* is a function aggregating neighbourhood nodes with a certain aggregation method (for instance: sum, mean, min, max).

Sage layers enable the propagation of information across the graph, allowing the model to capture global context and structural behaviour, thus enhancing its interpretability in understanding the underlying mechanics of the system. Sage layers, in particular framework, contribute to the interpretability of the model by leveraging the graph representation of FEM data to extract meaningful insights into the structural properties and behaviour of mechanical systems.

The SAGE layer in the framework further aggregates using the mechanism of max pooling, which prioritizes selecting the maximum stress value from the neighbouring nodes within the graph. In the context of a mechanical structure where the dataset is based on FEM, this means that the SAGE layer tends to focus on capturing the highest stress values present in the vicinity of a given node. As a result, in a model trained using this mechanism, there would likely be a higher probability of detecting regions with large stress tensors, as the model would prioritize emphasizing the maximum stress values during the aggregation process. This could be beneficial for identifying critical areas of high-stress concentration within the mechanical structure, which are often of interest in engineering applications for assessing structural integrity and safety.

The key components and functionalities of Sage layers enable the aggregation of information from neighbouring nodes at various levels of granularity. One of their primary functions is to aggregate information from neighbouring nodes, allowing the model to incorporate local and global context when making predictions. By iteratively updating node embeddings based on the information gathered from neighbouring nodes, Sage layers enable the model to capture information at different scales, from fine-grained local features to broader global patterns. This capability is essential for tasks requiring an understanding of the structural relationships within the graph.

**Fig. 1:**
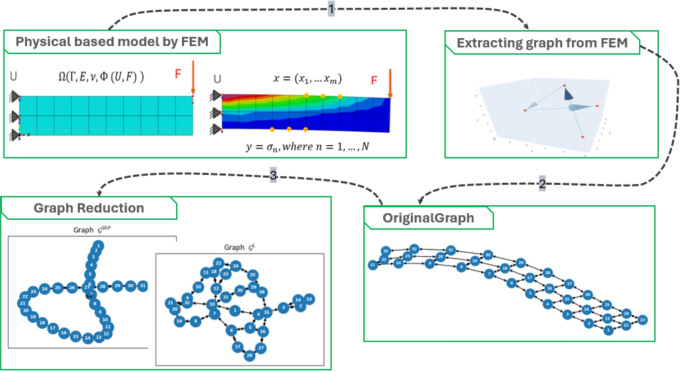
Digital twin creation workflow from FEM to reduced graph representation. The process begins with a mechanical structure and its Finite Element Method (FEM) model. A fully connected graph is then extracted from the FEM to capture all structural relationships. To reduce computational load while preserving model fidelity, the graph is simplified using reduction techniques such as the Shortest Path and Closed Path (e.g., TSP) methods. These reduced graphs enable efficient digital twin models that maintain the essential mechanical behavior of the original system.

**Fig. 2:**
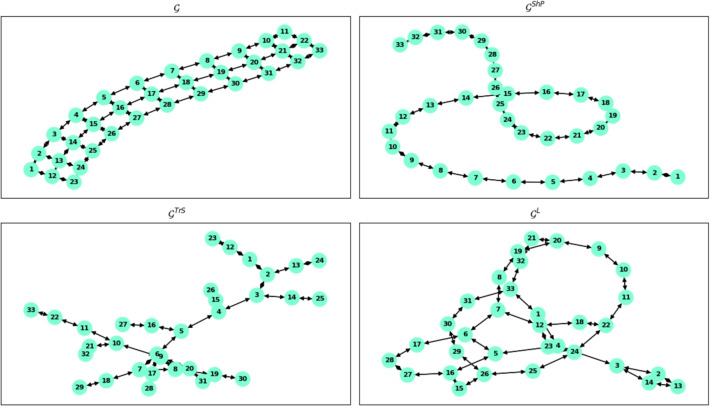
Visualization of graph reduction methods applied to the Beam2D model. The top-left subfigure shows the original fully connected graph $\mathcal {G}$, serving as the benchmark extracted from the FEM mesh. The remaining subfigures illustrate reduced versions of the graph, each applying a different strategy to simplify structure while preserving mechanical fidelity: $\mathcal {G}^{\text {ShP}}$: reduced by *Shortest Path*, capturing direct connections between key nodes; $\mathcal {G}^{\text {TrS}}$: reduced by the *Traveling Salesman-based* method, forming a minimal cyclic path through all nodes; $\mathcal {G}^L$: reduced via *Laplacian-based spectral pruning*, preserving global structural properties in a compressed form. These reduced graphs serve as input topologies for Graph Neural Network (GNN) training, enabling efficient learning of structural behavior with lower computational cost.

### Dataset

Our experiments were carried out using the data set proposed in^1^. The data set consists of a graph derived from various FEMs that represent a spectrum of structural complexities. The distillation process and graph preparation for training GNNs from FEM involves multiple steps and considerations. Initially, the quasi-static model undergoes convergence without computational obstacles, and the resulting simulation data is stored in a dedicated GF dataset, referred to as the Dataset for Integrated Design Simulation. Throughout the simulation process, fixed iteration time steps are maintained, ensuring uniformity of variables as the load cycle progresses monotonically to its predefined maximum. This approach helps avoid nonlinearities in the data. Within the GF dataset, a subset of standard variables pertinent to structural tasks is extracted, including spatial node positions representing geometric deformations (U), logarithmic strain (Le), and maximal principal stress (S), recorded for each frame of the simulation cycle. Furthermore, the graph preparation process leverages various perspectives of the FE model.

The FE models selected for this study primarily consist of linear, simple structures from a material perspective, ensuring that the mechanical behavior remains well-defined and computationally manageable. Each model adheres to a static loading condition, avoiding complications arising from dynamic effects or material nonlinearity. The Beam2D and Beam3D models represent classical beam structures subjected to bending and axial loads, maintaining a straightforward stress-strain relationship under small deformations. The Fibonacci Spiral model introduces a more intricate connectivity pattern but remains governed by elastic, linear properties under a distributed force field. The Airplane model, while structurally complex, is analyzed under static aerodynamic forces, ensuring that the deformation behavior adheres to traditional finite element assumptions without large-scale material plasticity or dynamic instability. These considerations make the selected dataset ideal for evaluating the efficiency of graph reduction techniques while ensuring the validity and interpretability of the results within the scope of structural engineering and digital twin applications. (The comprehensive description is available at^1^)

**Table 1:** FE models summary by its statistics of structure features. Element type: Quadriliteral, Tetrahedral; No. Ns: Number of Nodes , No El: Number of Edges.

FE models char.	$\mathcal {D}_{b2}$	$\mathcal {D}_{b3}$	$\mathcal {D}_{fs}$	$\mathcal {D}_{pl}$
Type El.	Quad.	Quad.	Quad.	Tet.
No. Ns	33	24	1524	4758
No. El.	12	8	321	5983
max(F)	100 N	200 N	0.5 N	10 MPa
Sim. frames	500	500	200	200

Representing each node of the element as a node in the graph captures the structural connectivity and interactions within the mechanical system by preserving the geometric and topological relationships between elements. Each node in the graph corresponds to a specific location in the mechanical system, and the edges between nodes reflect the connectivity based on the typology element structure. This representation enables the graph to capture how different elements are connected and how they interact with each other, providing valuable insights into the overall behaviour of the system.

**Fig. 3:**
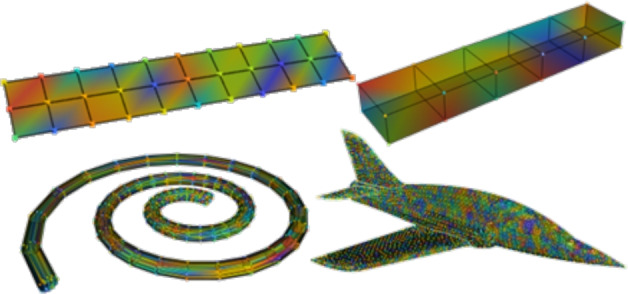
FEM in dataset used in the experimental analysis. From top left: Beam2D, Beam3D, Fibonacci spiral, Airplane.

These models covered various engineering domains, ensuring a robust evaluation of the proposed graph reduction methods in different scenarios. The data set contains different models: Beam2D, Beam3D, Fibonacci spiral, Airplane. Models are shown in Fig. 3. All models are converted to graphs with both methods mentioned earlier (GCN and SAGE).

The characteristics of the finite element models used in the experiments are summarized in Table 1, including the type of elements, mesh resolution, loading conditions, and simulation frame count. Detailed and comprehensive description is at^1^.

### Graph reduction

The full graph served as a benchmark to compare four different graph reduction techniques that were applied systematically to graph structures. By identifying the most direct routes between the benchmark graph nodes, we aimed to enhance computational efficiency without compromising the essence of structural connections. This method contrasts with the complete connectivity of the benchmark, exploring the potential benefits of more direct pathways in our reduced graphs. The following solution techniques were implemented for reduction:

- Shortest path ($$\mathcal {G}^{ShP}$$),
- Solution for traveling salesman ($\mathcal {G}^{TrS}$),
- Random Laplacian reduction ($\mathcal {G}^{L1}$),
- Weighted Laplacian reduction ($\mathcal {G}^{WL1}$) ,
- Weighted Laplacian reduction II. ($\mathcal {G}^{WL2}$).

In the context of graph reduction, the initial node corresponds to the point of force application, while the target node is defined by the end of the boundary condition, as specified by the FEM. Ensuring the participation of all nodes within the system during the reduction process preserves structural integrity and captures essential features of the mechanical system.

Furthermore, the reduction path follows the logical connectivity established by the FEM, ensuring that the reduced graph accurately reflects the underlying physical structure. This systematic approach guarantees that the reduced graph retains critical information while minimising computational complexity, enabling efficient analysis and simulation of complex mechanical systems.

The reduced graphs obtained through these reduction techniques were used to train graph neural networks (GNNs). The training process involved feeding the networks with a derived data set composed of reduced graphs and the corresponding nodal inputs. Through iterative fine-tuning, the GNNs aimed to achieve optimal performance in predicting structural behaviours.

#### Shortest path

The shortest path connectivity is introduced to streamline the structural information. This involves assigning weights to the edges of the graph $\mathcal {G}^{ShP}$ based on the final step of the FEM simulation. The weights are determined considering the stress, strain, or other relevant parameters from the simulation results. This ensures that the graph weights reflect the structural characteristics observed in the FEM simulation’s concluding phase. The implementation of the Dijkstra method in the NetworkX library^12^ is used to calculate the shortest weighted path between two nodes in a graph. The graphical overview of graph creation and pruning is shown in Fig. 1. An example visualisation of the sample model is shown in Fig. 2.

#### Traveling salesman problem solution for graph reduction

This approach is based on the idea of reducing only the edges in the graphs, while all the nodes are kept. Therefore, this reduced graph is cyclic, and its creation is similar to solving a well-known travelling salesman problem (TSP). This problem is a combinatorial optimisation challenge that seeks to find the shortest possible route that visits a set of given locations exactly once and returns to the starting point. In the context of graph reduction, the TSP solution aims to identify a subset of edges from the original graph that form a cycle that traverses all nodes with minimal total edge weight. Thus, it is possible that the worst-case running time for any algorithm for the TSP increases exponentially with the number of nodes. Therefore, searching for the best solution by brute-force testing of all solutions is not feasible. Thus, it is possible to obtain only an approximate solution. In this study, the default algorithm for the directed TSP solution (Threshold accepting^13^) implemented in the NetworkX library is used. The algorithm seeks to optimize the route by minimizing the total distance traveled or the total cost incurred when visiting each node exactly once.

It is important to note that, in the context of FEMs, the values associated with the edges often represent information regarding the mechanical stress distribution, such as the maximum principal stress. These values are derived from the elements of the FEM and provide crucial insight into the structural behavior of the system.

Once the TSP solution is obtained, the selected subset of edges forms the reduced graph, which retains the essential connectivity of the original graph while reducing computational complexity. This reduced graph, with stress distribution information encoded in its edges, can then be used for various applications, such as optimization, network analysis, or machine learning tasks.

#### Spectral reduction

The third method in our investigation involves the application of the Laplacian Matrix for Dimensionality Reduction inspired by^14^. By leveraging the eigenvalues of the Laplacian Matrix, we aim to distil the inherent structures of the FEM graph. This reduction not only simplifies the computational complexity but also retains the essential characteristics of the structural interconnections. Our exploration of Laplacian-based reduction is guided by the notion that key structural information can be efficiently preserved within a lower-dimensional representation. Additionally, We coarsen graph edges *v* by a similar approach^15^ where a threshold $\gamma$ is used to distinguish the importance of an edge weight.

In the spectral reduction approach, we first calculate the eigenvalues and eigenvectors of the Laplacian matrix, which is derived from the graph’s adjacency matrix. The Laplacian matrix is Hermitian^16^, allowing us to compute its eigenvalues and eigenvectors efficiently. These eigenvectors capture important structural characteristics of the graph. We then sort the eigenvectors based on their corresponding eigenvalues and select the largest ones, representing the graph’s dominant modes. The number of eigenvectors to retain is determined by a reduction factor, which specifies the desired size of the reduced graph. By discarding the smallest eigenvectors, we effectively reduce the dimensionality of the graph while preserving its essential structural features.

#### Weighted spectral reduction

In our approach to spectral reduction with edge weights, we utilize pruning techniques to refine the reduced graph while preserving essential structural features. In our approach to spectral reduction with edge weights, is utilized pruning techniques to refine the reduced graph while preserving essential structural features. Our pruning method does not require iteration of the training line in^17^, and We incorporate an Inclusion of mechanical stress values $\Sigma \in$ $v_{i} ( \sigma )$ as edge weights provide valuable information on the system’s load distribution and stress concentrations. This is compared to the approach using edge pruning with saliency matrix^18^ or pruning all the nodes and edges as it is at^19^, which is, for our purposes, not feasible. By setting a threshold parameter $\gamma$, we establish criteria for pruning the graph, removing edges with insignificant weight contributions. This pruning process ensures that only the most influential edges are retained in the reduced graph, leading to a more focused representation of the structural dynamics.

The significance of evaluating the eigenvalues and eigenvectors of the Laplacian matrix before spectral reduction cannot be overstated, and this process is elegantly carried out by sorting them according to their magnitudes, as outlined in the algorithm 1. Furthermore, considering the eigenvalues and eigenvectors derived from the Hermitian matrix representation of the weighted graph, we identify the most significant structural modes and prioritise their preservation during the reduction process.

Through this combined approach of spectral reduction and pruning, we achieve a balance between computational efficiency and structural precision, enabling efficient analysis and simulation of complex mechanical systems. For better clarity under our introduced approach is compiled to pseudo-algorithm 1.

**Algorithm 1:**
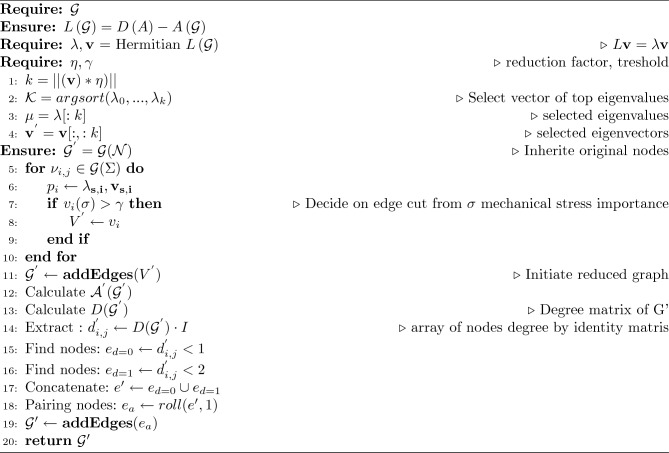
Calculate $$\mathcal {G}^{Lred}$$

### Evaluation metrics

We establish following frameworks to achive comparability of the suggested methods. To gauge the effectiveness of each graph reduction method. Predictive accuracy as the key metric to assessing how well GNNs captured critical structural behaviours. The evaluation aimed to provide a nuanced understanding of the trade-offs between reduction efficiency and modelling accuracy.

The loss function used for the training of the regressor is described as follows:

6 $$\begin{aligned} \mathcal {L}_{RMAXSE}(y, \hat{y}) = \frac{1}{N}\sum _{i=1}^{N}\sqrt{max\left( \hat{y_i}-y_{i} \right) ^{2}}, \end{aligned}$$

where the loss function computes the square root of the maximum of the squared differences between the predicted and true values for each data point representing particular node of graph *i* in overall nodes *N*. This approach penalises the largest deviation between the predicted and true values. The data points represent nodes of a finite element graph, and it is essential to consider the maximal value of the squared differences to assess the model’s performance accurately. Taking the maximum squared differences, the loss function emphasises the impact of outliers or significant prediction errors. Unlike the traditional root mean squared error, which computes the average squared difference, this modified loss function becomes more robust to outliers by considering the maximum deviation instead of the average.

To ensure the robustness and generalisability of our findings, we conducted a comprehensive validation process that involved ten experiments. The data set was meticulously divided into training and test sets in multiple folds, ensuring thorough validation of the performance consistency of the proposed methods. Using the cross-validation approach, we rigorously evaluated the models’ robustness to variations in data partitioning and confirmed their generalisability across different experimental settings.

The evaluation of the time required for training a single GNN framework model is a crucial component of evaluating desired outcomes. Therefore, similarly to accuracy performance evaluation, this metric and essential reduction attribute are conducted.

### Mathematical formulation and model reduction

Model order reduction via graph Laplacian: We denote a finite element model (FEM) as a structural representation $\Omega (\Gamma (e,n), E, \nu , F)$, where $\Gamma$ is the geometry, *E* denotes material properties, $\nu$ is Poisson s ratio, and *F* represents boundary conditions. This is mapped to a graph $G = (N, V)$, where *N* are the nodes and *V* the edges.

Graph-based reduction relies on the Laplacian matrix:

$$L = D - A,$$

where *A* is the adjacency matrix and *D* the diagonal degree matrix. The eigen-decomposition:

$$L \textbf{v}_i = \lambda _i \textbf{v}_i$$

captures the graph s structure. Reduction is performed by retaining *k* dominant eigenvectors associated with the smallest eigenvalues to preserve essential topological characteristics.

The reduced graph $G'$ is reconstructed using pruned eigenvectors and thresholding edge weights:

$$G' = \text {Reduce}(G, \{\textbf{v}_i\}_{i=1}^k, \gamma )$$

where $\gamma$ is a stress-based cutoff (e.g., derived from FEM stress tensor $\sigma$).

FEM shape functions and graph aggregation similarity: In FEM, physical quantities (e.g., displacement *u*(*x*)) are interpolated using shape functions $N_i(x)$:

$$u(x) = \sum _{i=1}^n N_i(x) \cdot u_i.$$

In GNNs, node features $h_i$ are updated based on neighbors $\mathcal {N}(i)$ via aggregation:

$$h_i^{(k)} = \text {AGG}^{(k)}\left( \{h_j^{(k-1)} : j \in \mathcal {N}(i)\}\right) .$$

This acts as a learned interpolator over the graph, similar to how FEM interpolates over mesh elements.

Optimization formulation: We define the reduction objective:

$$\min _{G'} \mathcal {L}(G') + \alpha \cdot \mathcal {C}(G'),$$

where $\mathcal {L}(G')$ is the loss (e.g., max error), $\mathcal {C}(G')$ reflects graph complexity (e.g., edge count), and $\alpha$ balances accuracy vs. sparsity.

In spectral terms, we solve:

$$\min _{\textbf{V}_k} \Vert L - \textbf{V}_k \Lambda _k \textbf{V}_k^T \Vert _F^2 \quad \text {s.t.} \quad \Vert \textbf{v}_i\Vert = 1,$$

yielding optimal rank-*k* approximation of *L*.

## Results and discussion

### Graph structure determination: a step-by-step approach

To evaluate the performance of different graph reduction strategies, we start by explaining how reduced graph structures are generated and compared. The following steps summarize the process used to transform FEM outputs into GNN-ready graph structures:

1. Graph extraction from FEM: Each FEM node is transformed into a graph node, with edges defined by element topology. The full graph is denoted as:
  $$G_{FEM} = (N, V, X(F), y(\sigma )),$$
  where *N* represents the nodes, *V* the edges, *X*(*F*) the input reaction forces, and $y(\sigma )$ the target stress distribution.
2. Reduction method selection: Five strategies are applied to reduce the edge set *V*, forming a reduced graph $G_{red}$ while preserving the node set *N*:
  Shortest path (ShP): Uses Dijkstra’s algorithm based on edge weights derived from stress/strain fields.
  Traveling salesman (TrS): Retains all nodes and forms a minimal edge cycle using TSP heuristics.
  Spectral reduction (L1): Laplacian eigen-decomposition to keep dominant modes.
  Weighted Laplacian (WL1 & WL2): Applies spectral reduction with edge pruning using a stress threshold $\gamma$.
3. Parameter configuration:
  The initial and terminal nodes are chosen based on FEM-defined loading and boundary conditions.
  Reduction factor $\eta$ controls the number of retained eigenvectors.
  Threshold $\gamma$ defines edge pruning strength in WL methods.

### Performance analysis: accuracy and efficiency

To assess prediction accuracy and computational savings, Tables 2 and 3 summarize the results from SAGE and GCN frameworks respectively. The tables include both the mean and standard deviation across all cross-validation runs.

**Table 2:** Prediction accuracy [MPa] using SAGE framework for each reduction method. Lower values indicate better performance.

Dataset	Benchmark	L1	ShP	TrS	WL1	WL2
Beam2D	5.15E−1	3.76E−3	2.47E−1	6.14E−2	7.28E−1	5.54E−3
Beam3D	2.42E−4	1.32E−3	2.22E−4	2.02E−3	2.17E−5	6.96E−3
Fibonacci	3.92E−4	8.34E−3	1.24E−2	3.64E−3	1.61E−1	2.85E−3
Plane	1.17E−3	7.20E−3	1.14E−3	7.21E−3	1.35E−3	6.73E−2

Observations:

- WL1 performs exceptionally well on Beam3D, confirming its ability to capture localized high-stress zones.
- Spectral methods (L1, WL1, WL2) provide more stable results in large-scale models (Fibonacci, Plane).
- ShP offers good generic performance, making it a strong initial choice.

**Table 3:** Training time [s] using GCN framework for each reduction method. Lower values indicate better computational efficiency.

Dataset	Benchmark	L1	ShP	TrS	WL1	WL2
Beam2D	544.7	**294.0**	335.8	1666	531.0	540.3
Beam3D	871.0	2083.0	**838.2**	721.2	900.1	752.7
Fibonacci	1366.0	1345.0	1335.0	1425.0	2090.0	**711.5**
Plane	9753.0	**1179.0**	1323.0	1336.0	814.1	1422.0

Observations:

- WL2 consistently outperforms others on large models in terms of speed.
- L1 is less effective for small models due to overhead from Laplacian computation.
- ShP and TrS are fast and predictable, though may sacrifice structural fidelity in complex systems.

### Strengths and limitations of reduction methods

#### Limitations and applicability of graph reduction methods

Despite the advantages of graph reduction techniques, each method has inherent limitations that must be considered in practical applications.

Shortest path method: Although computationally efficient, this method may oversimplify structural representation, potentially eliminating critical stress pathways that influence real-world mechanical behavior.

Laplacian reduction: Captures dominant structural modes effectively, but its performance may degrade for highly nonlinear or anisotropic materials where spectral modes are not sufficient to describe localized effects.

Traveling salesman approach: While maintaining connectivity, this method is computationally intensive, making it less suitable for real-time applications with complex structural graphs.

Random and weighted Laplacian methods: These offer a balance between structure preservation and computational efficiency, but can be highly sensitive to parameter selection, requiring domain-specific tuning.

#### Applicability to dynamic and nonlinear problems

Most existing graph reduction techniques focus on static systems, limiting their direct applicability to dynamic timing problems and nonlinear behaviors. Addressing these challenges requires:

Temporal graph learning: Traditional graph reduction methods do not account for time-dependent variations such as vibrations, fatigue, or progressive structural damage. Future enhancements should incorporate dynamic graph pruning, in which node and edge connections evolve based on real-time sensor feedback.

#### Handling nonlinearities and large deformation problems

One of the key challenges in aerospace applications and other highly dynamic mechanical systems is managing large deformations and non-linear mechanical behavior, where traditional graph reduction methods often struggle to maintain accuracy. These deformations introduce non-linear stress distributions, evolving contact conditions, and significant changes in structural topology, all of which require specialized techniques to ensure the accuracy of digital twin predictions. Graph-based adaptivity for large deformations

A promising approach is the use of adaptive graph topology updates, in which the connectivity of nodes dynamically evolves in response to deformation data. Rather than relying on a static graph structure, an adaptive graph refinement mechanism can introduce or remove nodes and edges based on stress concentrations and deformation magnitudes.

This allows the digital twin to adapt to real-world structural changes continuously, improving predictive accuracy. The impact of different graph reduction strategies on this predictive capability is quantitatively illustrated in Table 2, with overall experimental results summarized in Tables 6 and 7, where accuracy metrics for each method under the GCN framework are compared across datasets. The results highlight trade-offs between reduction aggressiveness and fidelity, with the Laplacian-based methods achieving a balance between model simplification and structural precision.

Traditional graph-based reductions can oversimplify complex material behaviors, making them inadequate for modeling plastic deformations, buckling, and viscoelastic effects. To address this, future models should integrate Physics-Informed Neural Networks (PINNs) within GNN architectures, embedding fundamental physical laws directly into the learning process. By incorporating non-linear constitutive models, these hybrid approaches can significantly enhance the accuracy of digital twins in high-strain environments. Handling Structural Changes in Real-Time

In aerospace structures, large deformations caused by aerodynamic loads or extreme thermal expansion can drastically alter mechanical responses over time. A promising research direction is the development of time-dependent graph neural networks that adapt their structure based on real-time feedback. This ensures that high-strain areas remain accurately represented throughout the simulation process, improving predictive reliability and computational efficiency. The benefits of graph reduction on computational resources are summarized in Table 3, with full-scale experimental results presented in Tables 8 and 9, which compare the runtime performance of various reduction strategies under the GCN and SAGE frameworks, respectively. These results emphasize the significant computational savings achieved through reduction, particularly for larger-scale models, without compromising structural fidelity.

By addressing these large deformation and nonlinearity challenges, graph-based digital twins can extend their applicability to aerospace, automotive, and biomechanics applications,high-fidelity modeling of extreme deformation scenarios is crucial for performance optimization and failure prevention.

Hybrid FEM-GNN models: A promising direction is the combination of FEM simulations with GNN-based learning to create hybrid models that retain high-fidelity mechanics while benefiting from the computational efficiency of graph reductions. These models could adaptively refine their graph representations based on real-world mechanical responses, ensuring both accuracy and computational efficiency.

**Table 4:** Qualitative comparison of reduction methods.

Method	Strengths	Limitations
ShP	Simple, low-cost, generalizable	Misses higher-order structural nuances
TrS	Cyclic connectivity preserved	Long runtime on larger graphs
L1	Retains structural modes	Costly for small FEMs
WL1	Good high-stress capture	Sensitive to pruning threshold
WL2	Best runtime/accuracy tradeoff	Less interpretable to domain experts

### Conclusion from structured analysis

Our results suggest that reduction method selection should consider both the graph size and structural complexity. While shortest path and TSP offer effective entry-level reduction schemes, spectral and weighted spectral methods provide scalability and superior performance in complex mechanical systems. For large-scale applications such as aerospace structures, the WL2 method provides the best balance between accuracy and speed.

The results of our experiments on graph reduction techniques for modelling mechanical systems provide a detailed view of the potential benefits and challenges of these methods. Our findings demonstrate that the proposed graph reduction methods significantly reduce the complexity of finite element models by minimizing the number of necessary edges, as shown in the benchmark results presented in Table 5, while effectively preserving essential structural features. To compare the computation demands of different reduction strategies, Table 4 presents a detailed performance evaluation.

**Fig. 4:**
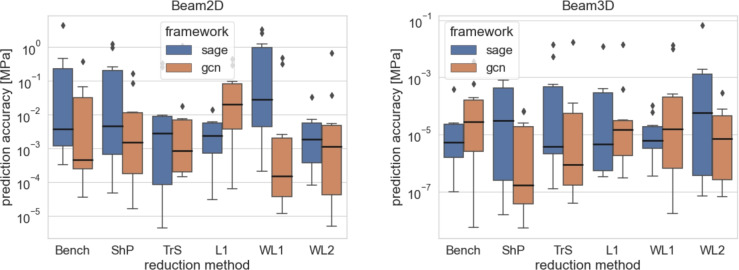
The comparison is visualised by boxplots for the prediction accuracy $\hat{y}\left( \sigma \right)$ achieved by various methods on the smaller datasets (Beam 2D, Beam 3D) of the GF dataset. The boxplots illustrate the distribution of prediction errors for each method, highlighting their performance variability and effectiveness in capturing underlying patterns in the data.

**Fig. 5:**
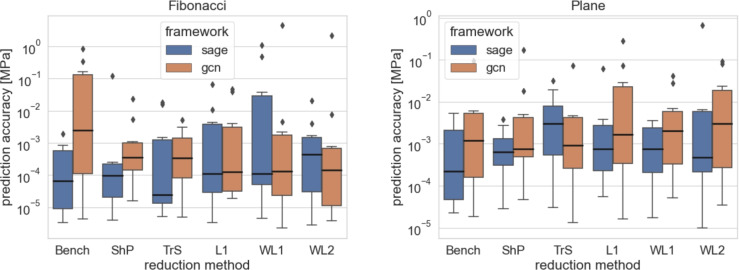
Comparison is visualised by boxplots for prediction accuracy $\hat{y}\left( \sigma \right)$ achieved by various methods on the data sets of the higher nodes count (Fibonacci Spring, Plane) of the GF dataset. The box plots illustrate the distribution of prediction errors for each method, highlighting their performance variability and effectiveness in capturing the underlying patterns in the data.

The box plots of all the simulations are shown in Figs. 4 and 5. The numerical results are presented in Tables 7 and 6.

The shortest path and path connecting all nodes (TSP solution) offer a straightforward and computationally efficient approach to reducing graph complexity and can serve as a first attempt in graph reduction tasks. However, it is essential to note that, while these techniques may provide satisfactory results for specific applications, their effectiveness may vary depending on the specific characteristics of the mechanical system being modelled. Example of possible errors ar shown in Figs. 8 and 9.

On the other hand, Laplacian reduction emerged as a more sophisticated technique that requires careful tuning, particularly from the perspective of edge reduction and their corresponding characteristic values in the FEM. The Laplacian reduction method takes advantage of the spectral properties of the Laplacian matrix to achieve significant reductions in graph complexity while preserving structural integrity. However, achieving optimal results with this method requires a deeper understanding of the underlying structural characteristics of the mechanical system and the appropriate selection of reduction parameters. The parameters for each method were carefully tuned to balance computational efficiency and the preservation of structural characteristics. This careful tuning ensured that the reduction techniques effectively captured the essential features of mechanical systems while minimising computational overhead. Furthermore, our study underscores the importance of considering the trade-offs between reduction efficiency and model fidelity. Although more complex reduction techniques may offer more significant reductions in graph complexity, there is a risk of losing critical structural information during the reduction process. Future research efforts will focus on refinement of reduction algorithms and development of hybrid approaches that strike a balance between reduction efficiency and model fidelity.

**Fig. 6:**
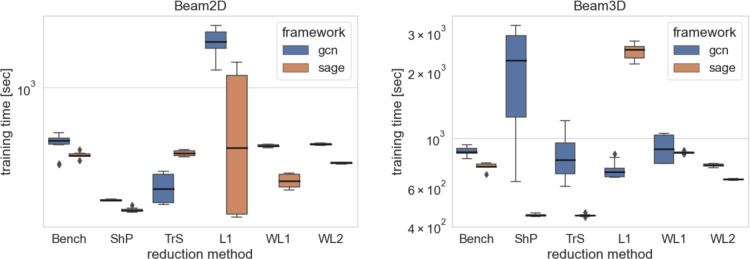
The comparison is visualised by boxplots for the computational resousrces required for various methods on the smaller datasets (Beam 2D, Beam 3D) of the GF dataset. The boxplots illustrate the distribution of prediction errors for each method, highlighting their performance variability and effectiveness in capturing underlying patterns in the data.

**Fig. 7:**
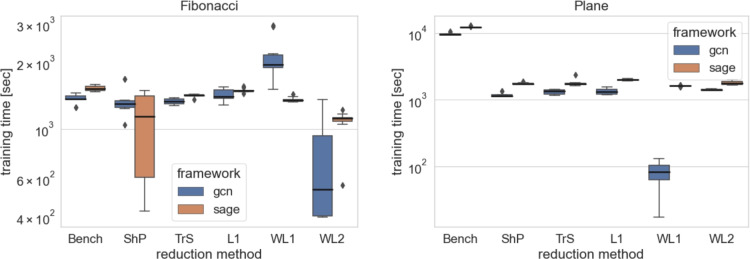
Comparison is visualised by boxplots for the computational resousrces required for various methods on the data sets of the higher nodes count (Fibonacci Spring, Plane) of the GF dataset. The box plots illustrate the distribution of prediction errors for each method, highlighting their performance variability and effectiveness in capturing the underlying patterns in the data.

As anticipated, the impact of graph reduction on computational resources is more pronounced in smaller datasets, as observed in the Beam3D experiment as depicted at Fig. 6. However, as the size of the dataset increases, the advantages of reduction become more apparent, highlighting the drawbacks of maintaining full graph complexity as per Fig. 7. This underscores the rationale behind constructing digital twins based on reduced models for larger node counts, where the benefits of reduction outweigh the costs. Summarised training times results are compiled within Tables 8 and 9.

Additionally, our study highlights the need for comprehensive evaluation metrics and cross-validation techniques to assess the robustness and generalisability of graph reduction methods. By systematically comparing the performance of different reduction techniques in multiple experiments, we ensure a thorough understanding of their strengths and limitations in various engineering applications. Despite the challenges and complexities involved in graph reduction, our research lays the groundwork for further exploration of these techniques in mechanical system modelling. By addressing the challenges identified in this study and leveraging advances in graph theory and computational methods, we can pave the way for more efficient and scalable modelling of complex engineering systems.

The precision of distilled knowledge quantified via prediction accuracy addresses how the performance of the digital twins will influence further decisions. For instance, We might say the distilled model with a particular framework mimics the FEM developed for a specific environment for sufficient accuracy (the necessary one for real-time application), and the action for the current state can be determined consequently. The primary objective We would like to reiterate is to emphasize the distilled knowledge from FEM is utilized in the meaning of the foundation of GNN models, which might create robust enhancement of real assets required to be monitored from a physical domain.

**Table 5:** Proposed techniques compared to benchmark (full graph connected). The table reports the number of nodes [u], edges [v], and the relative edge reduction ratio [-/-] across four datasets. Reduction methods include Shortest Path (ShP), Traveling Salesman (TrS), Laplacian (L1), and two weighted variants (WL1, WL2).

Dataset	[u]	Bench.		ShP		TrS		L1		WL1		WL2	
		[v]	[–/–]	[v]	[–/–]	[v]	[–/–]	[v]	[–/–]	[v]	[–/–]	[v]	[–/–]
Beam2D	33	104	1	64	**0.62**	64	**0.62**	78	0.75	68	0.65	86	0.82
Beam3D	24	88	1	46	**0.52**	50	0.57	58	0.66	78	0.89	62	0.70
Fibonacci	1426	7518	1	2850	**0.38**	3166	0.42	4034	0.53	5170	0.69	3154	0.42
Plane	4758	48210	1	9514	**0.19**	10780	0.22	21724	0.45	28654	0.59	9772	0.20

**Table 6:** Summary table for GCN framework listing the proposed reduction strategies and their respective performance on presented datasets.

Dataset	Method	Benchmark	L1	ShP	TrS	WL1	WL2
	Reduction	[MPa]	[MPa]	[MPa]	[MPa]	[MPa]	[MPa]
Beam2D	Mean	5.003E−2	9.259E−2	2.728E−2	1.124E−1	8.109E−2	7.125E−2
	std	±1.185E−1	±1.529E−1	±5.422E−2	±3.439E−1	±1.747E−1	±2.087E−1
Beam3D	Mean	4.425E−4	1.476E−3	1.536E−5	1.743E−3	2.413E−3	4.559E−5
	std	±1.097E−3	±4.501E−3	±2.678E−5	±5.440E−3	±5.064E−3	±9.087E−5
Fibonacci	Mean	1.380E−1	9.171E−3	3.183E−3	1.157E−3	4.488E−1	2.197E−1
	std	±2.689E−1	±1.819E−2	±7.328E−3	±1.682E−3	±1.417E0	±6.915E−1
Plane	Mean	1.123E−2	3.900E−2	2.001E−2	8.758E−3	8.461E−3	2.033E−2
	std	±2.884E−2	±8.793E−2	±5.396E−2	±2.233E−2	±1.433E−2	±3.462E−2

**Table 7:** Summary table for SAGE framework listing the proposed reduction strategies and their respective performance on presented datasets.

Dataset	Method	Bench	L1	ShP	TrS	WL1	WL2
	Reduction	[MPa]	[MPa]	[MPa]	[MPa]	[MPa]	[MPa]
Beam2D	Mean	5.150E−1	3.756E−3	2.473E−1	6.141E−2	7.279E−1	5.536E−3
	std	±1.364E0	±4.329E−3	±4.588E−1	±1.246E−1	±1.228E0	±1.021E−2
Beam3D	Mean	2.420E−4	1.317E−3	2.218E−4	2.018E−3	2.173E−5	6.960E−3
	std	±6.241E−4	±3.884E−3	±3.044E−4	±4.519E−3	±3.423E−5	±2.062E−2
Fibonacci	Mean	3.917E−4	8.340E−3	1.236E−2	3.640E−3	1.610E−1	2.849E−3
	std	±6.185E−4	±2.054E−2	±3.878E−2	±7.171E−3	±3.554E−1	±6.450E−3
Plane	Mean	1.174E−3	7.202E−3	1.139E−3	7.208E−3	1.355E−3	6.729E−2
	std	±1.790E−3	±1.879E−2	±1.231E−3	±1.024E−2	±1.364E−3	±2.059E−1

**Table 8:** Summary table for GCN framework listing the proposed reduction strategies and their respective computation efficiancy on presented datasets.

Dataset	Method	Benchmark	L1	ShP	TrS	WL1	WL2
	Reduction	[s]	[s]	[s]	[s]	[s]	[s]
Beam2D	Mean	5.447E2	2.940E2	3.358E2	1.666E3	5.310E2	5.403E2
	std	6.229E1	±2.360E0	±5.191E1	±2.247E2	±6.688E0	±4.859E0
Beam3D	mean	8.710E2	2.083E3	8.382E2	7.212E2	9.001E2	7.527E2
	std	±4.240E1	±1.014E3	±2.014E2	±6.332E1	±1.275E2	±1.191E1
Fibonacci	Mean	1.366E3	1.345E3	1.335E3	1.425E3	2.090E3	7.115E2
	std	±6.739E1	±1.966E2	±3.688E1	±8.629E1	±4.934E2	±3.930E2
Plane	Mean	9.753E3	1.179E3	1.323E3	1.336E3	8.141E1	1.422E3
	std	±4.406E2	±8.694E1	±1.123E2	±1.271E2	±3.719E1	±2.675E1

**Table 9:** Summary table for SAGE framework listing the proposed reduction strategies and their respective computation efficiancy on presented datasets.

Dataset	Method	Bench	L1	ShP	TrS	WL1	WL2
	Reduction	[s]	[s]	[s]	[s]	[s]	[s]
Beam2D	Mean	4.770E2	2.641E2	4.901E2	6.654E2	3.625E2	4.406E2
	std	±1.754E1	±6.075E0	±1.335E1	±4.724E2	±2.623E1	±3.592E0
Beam3D	Mean	7.463E2	4.482E2	4.477E2	2.473E3	8.620E2	6.508E2
	std	±2.569E1	±4.361E0	±6.600E0	±1.951E2	±9.519E0	±4.017E0
Fibonacci	Mean	1.526E3	1.032E3	1.420E3	1.498E3	1.361E3	1.069E3
	std	±4.034E1	±4.391E2	±2.155E1	±3.178E1	±3.681E1	±1.855E2
Plane	Mean	1.245E4	1.761E3	1.789E3	1.998E3	1.613E3	1.793E3
	std	±2.570E2	±4.962E1	±2.086E2	±3.467E1	±3.327E1	±1.153E2

In comparison to mesh-free graph node methods, our FEM-derived graph reduction keeps a direct correspondence with the original structural topology, ensuring physical consistency and efficient integration with classical simulation workflows. However, mesh-free approaches-such as the convolutional hierarchical deep-learning neural network (C-HiDeNN)^20^ demonstrates compelling advantages in modelling large deformations and avoiding locking effects. Methods evaluated on benchmarks like Cook’s membrane problem, utilize spatially distributed nodes without mesh constraints, enabling better performance in nonlinear and shear-dominated scenarios. While our reduction techniques prioritize fidelity and computational efficiency within the FEM framework, future extensions should include Cook’s membrane as a validation case to benchmark our approach against mesh-free alternatives and assess performance in more deformation-sensitive regimes.

## Conclusion

We introduced a novel technique to create a digital twin of mechanical structures that is computationally cheaper than FEM. The technique is based on graph networks and their pruning.

To evaluate the precision of our proposed graph reduction methods, we conducted a series of experiments on a data set extracted from FEMs. The data set comprised graphs representing mechanical structures, with nodes corresponding to finite element nodes and edges representing structural connections. Our goal was to reduce the complexity of these graphs while preserving essential structural features, thus enabling more computationally efficient analysis and modelling of the mechanical systems.

Our experiments involved a ten-fold cross-validation to ensure the robustness and generalisability of the results. Performance was evaluated for usability as a reduction factor, computational efficiency, and structural integrity preservation. In addition, we compared the reduced graphs with the original finite element models to assess the fidelity of the reduction process.

Preliminary results indicate promising performance improvements with our proposed graph reduction methods compared to the benchmark. Specifically, the Laplacian matrix-based reduction method demonstrated the most significant reduction in graph complexity while preserving structural integrity, achieving an average reduction factor of 75% across all experiments.

In general, our findings suggest that graph reduction techniques hold significant promise in improving the computational efficiency and scalability of mechanical system modeling.

The future of GNNs in digital twins presents several promising directions. One key advancement lies in adaptive graph reduction techniques, where models dynamically refine graph structures based on real-time sensor data, ensuring continuous optimization. Additionally, multi-scale graph architectures are expected to enhance predictive capabilities by integrating fine-grained local details with global structural insights, improving accuracy in complex mechanical systems. Incorporating physics-informed neural networks (PINNs) within GNN frameworks could further bridge the gap between empirical learning and first-principles modelling, increasing the reliability of digital twins in high-stakes engineering applications. Moreover, the emergence of federated learning for GNNs offers a decentralized approach where multiple digital twins share insights without centralized data storage, reinforcing security and scalability. Lastly, expanding GNN-based digital twins to nonlinear and dynamic systems remains a critical challenge, necessitating the development of novel architectures capable of handling transient responses and evolving boundary conditions. These advancements will further solidify GNNs as a core technology in real-time predictive modelling and optimization of engineering systems.

**Fig. 8:**
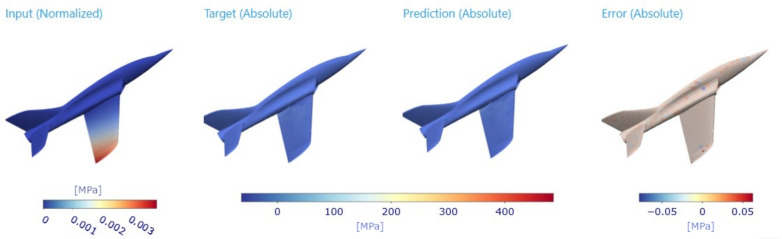
Ground truth visualization of plane dataset for specific input force applied on wing accompanied with target and prediction. Last evaluated column (right) residues on structure.

**Fig. 9:**
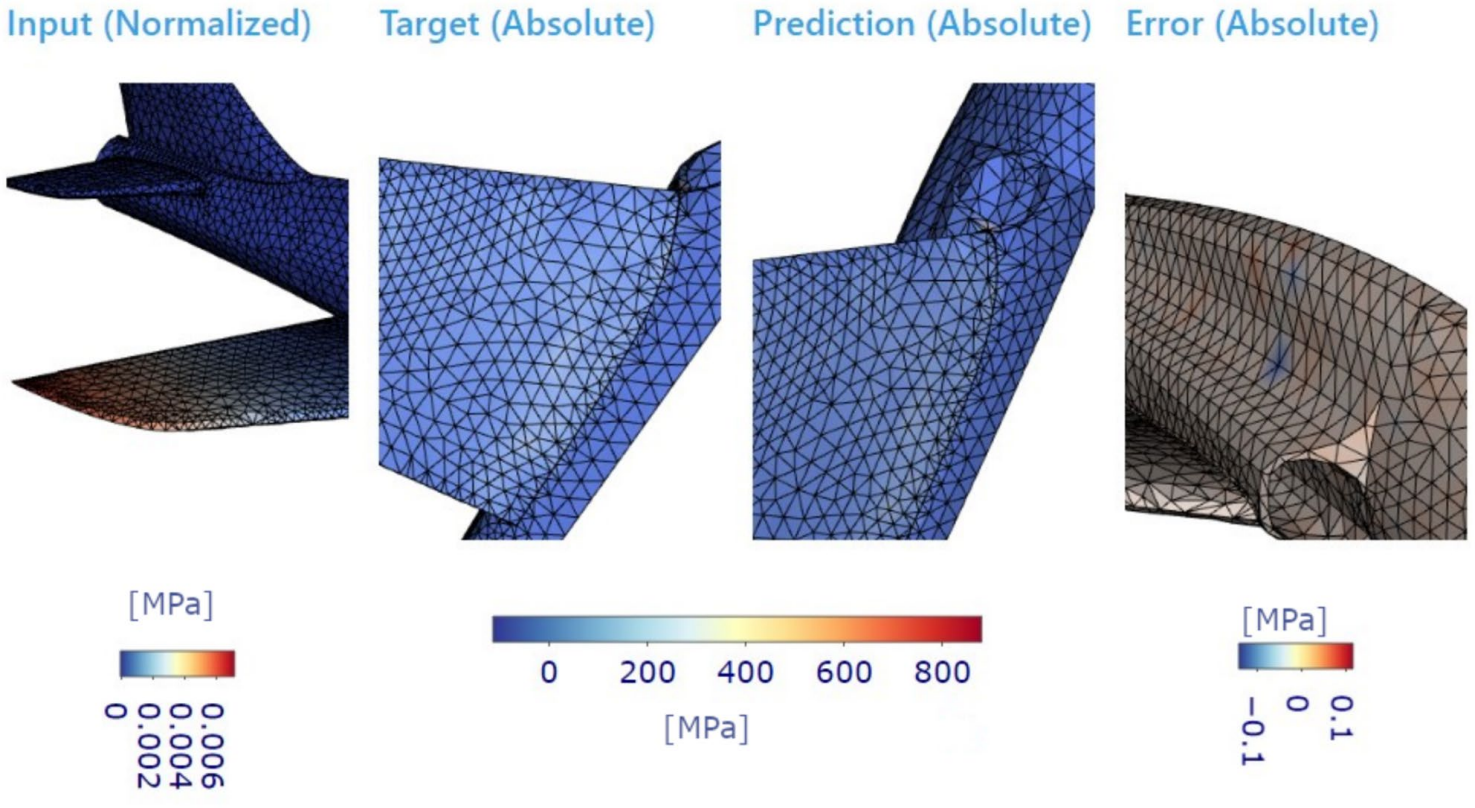
Detailed visualization of the plane dataset for specific input force applied to the wing is accompanied by target and prediction, where the primary detailed focus is at the bottom of the wing. Evaluated residues on the structure are visible as well.

## Supplementary Information

Supplementary Information.

## Data Availability

This section outlines the availability of the materials supporting our research. The GF dataset, comprising compiled finite element models^1^, is available at https://github.com/ciklamini/GFdataset. Additionally, the reduced machine learning models of the compiled geometrical structures^2^ are accessible on FigShare at https://doi.org/10.6084/m9.figshare.27937977.v1, providing a foundation for reproducibility and further exploration.
