# Supplementary material for: Enhancing digital twin performance through optimizing graph reduction of finite element models
Source: Sci Rep. 2025 Oct 29;15:37777. doi: 10.1038/s41598-025-20571-z (PMC12572373; doi:10.1038/s41598-025-20571-z)
Supplement: Supplementary file 1 — Supplementary Information. [file 41598_2025_20571_MOESM1_ESM.pdf]

Appendices

Algorithm 2 Graph extraction from structure based on quadraliteral element

Ensure: $\mathcal{G} = Graph$

for nd_i in $nodes$ **do**
  $src \leftarrow nd_i$ ▷ source node
  $src_{pos} \leftarrow [src[x], src[y]]$
  $\mathcal{G} \leftarrow$ **addNode**(src_{pos})
end for
for i in $elements$ **do**
  $e_i \leftarrow nodes_i$
  $e_a i \leftarrow roll(e_i, 1)$
  $\mathcal{G} \leftarrow$ **addEdges**$(e_a i)$
end for
return $\mathcal{G}$

Algorithm 3 Graph extraction from structure based on tetrahedral element

Ensure: $\mathcal{G} = Graph$

for nd_i in $nodes$ **do**
  $src \leftarrow nd_i$ ▷ source node
  $src_{pos} \leftarrow [src[x], src[y], src[z]]$
  $\mathcal{G} \leftarrow$ **addNode**(src_{pos})
end for
for i in $elements$ **do**
  $e_i \leftarrow nodes_i$
  $e_a i \leftarrow reshape(e_i, 2, 2).T$
  $e_b i \leftarrow reshape(e_i, 2, 2)$
  $e_c i \leftarrow roll(e_i, 1)$
  $\mathcal{G} \leftarrow$ **addEdges**$(e_a i)$
  $\mathcal{G} \leftarrow$ **addEdges**$(e_b i)$
  $\mathcal{G} \leftarrow$ **addEdges**$(e_c i)$
end for
return $\mathcal{G}$

Algorithm 4 Graph extraction from structure based on hexagonal element

Ensure: $\mathcal{G} = Graph$

for nd_i in $nodes$ **do**
    $src \leftarrow nd_i$ ▷ source node
    $src_{pos} \leftarrow [src[x], src[y], src[z]]$
    $\mathcal{G} \leftarrow$ **addNode**(src_{pos})
end for
for i in $elements$ **do**
    $e_i \leftarrow nodes_i$
    $e_a i \leftarrow reshape(e_i, 2, 4).T$
    $e_b i \leftarrow reshape(e_{i(0:4)}, 2, 4)$
    $e_c i \leftarrow reshape(e_{i(4:end)}, 2, 4)$
    $e_d i \leftarrow roll(e_b i, 1)$
    $e_e i \leftarrow roll(e_c i, 1)$
    $\mathcal{G} \leftarrow$ **addEdges**$(e_a i)$
    $\mathcal{G} \leftarrow$ **addEdges**$(e_d i, e_e i)$
end for
return $\mathcal{G}$
